# Safety and Efficacy of a Plug‐Based Vascular Closure Device After Percutaneous Microaxial Flow Pump in the Treatment of Complex and High‐Risk Indicated Patients

**DOI:** 10.1002/ccd.31528

**Published:** 2025-04-10

**Authors:** Jörg W. Schröder, Norman Mangner, Felix J. Woitek, Kai U. Markus, Michael Lehrke, Felix Vogt, Marlo Verket, Alexandros Siskos, Moritz Biener, Benjamin Meder, Nicolas M. Van Mieghem

**Affiliations:** ^1^ Department of Cardiology, Angiology and Intensive Care University Hospital RWTH Aachen Aachen Germany; ^2^ Department of Internal Medicine and Cardiology, Heart Center Dresden University Hospital, Technische Universität Dresden Dresden Germany; ^3^ VYSYO GmbH, Clinical Research Eschweiler Germany; ^4^ Department of Cardiology Erasmus Medical Centre Rotterdam the Netherlands; ^5^ Department of Cardiology, Angiology and Pulmonology University Hospital Heidelberg Heidelberg Germany

**Keywords:** bleeding, CHIP, Manta, outcome, percutaneous microaxial flow pump, plug‐based vascular closure device

## Abstract

**Background:**

Femoral large‐bore vascular access is an important and constantly increasing technique in interventional treatment. Temporary circulatory support in complex and high‐risk indicated patients (CHIP) presents a challenge in terms of postinterventional access site closure.

**Aim:**

Monitoring the safety and efficacy of the MANTA system (Teleflex Inc.), a plug‐based vascular closure device (PbVCD) in patients receiving a percutaneous microaxial flow pump (mAFP).

**Methods:**

A multicenter prospective all‐comers registry enrolled 73 consecutive patients scheduled for PbVCD after receiving mAFP for CHIP or cardiogenic shock. The primary endpoint was the occurrence of vascular complications at the mAFP access site until discharge according to the Valve Academic Research Consortium‐3 (VARC‐3) definition. Secondary endpoints included the rate of procedure‐ or device‐related bleedings, device‐failures, and the time to hemostasis. Operators rated on the use of the MANTA System and the similarity compared to ANGIOSEAL (Terumo Medical Corp.).

**Results:**

Hemostasis was achieved in all 73 patients with no need for alternative treatment other than manual compression. The primary endpoint occurred in 7 (9.6%) patients with 1 (1.4%) patient suffering from a major vascular complication and 6 (8.2%) patients developing hematoma with type 2 bleeding (minor vascular complication). There was no vessel closure or thromboembolic event. In‐hospital survival was 100%.

**Conclusions:**

The PbVCD system is a safe, efficient and easy‐to‐use vascular closure system for large‐bore vascular access in patients receiving a mAFP. Advantages and disadvantages of PbVCD and suture‐based vascular closure devices (SbVCD) systems should be individually adapted according to the patient's medical history and the anatomy of the vascular constellation.

**Abbreviations**
CHIP: complex and high‐risk indicated patients
CS: cardiogenic shock
HRPCI: high risk percutaneous coronary intervention
mAFP: percutaneous microaxial flow pump
PAD: peripheral arterial vascular disease
PbVCD: plug‐based vascular closure device
SbVCD: suture‐based vascular closure devices
VARC‐3: Valve Academic Research Consortium‐3 consensus

## 1 Introduction

The need for a safe large‐bore access via the femoral artery for cardiovascular interventions has become increasingly important. The growing number of catheter‐based temporary circulatory support systems in complex and high‐risk indicated patients (CHIP) pose a challenge [1, 2]. Access methods have evolved from surgical exposure of the femoral artery with visual puncture of the vessel and postoperative surgical suture closure to ultrasound guided percutaneous vascular puncture and postoperative use of specialized vascular closure devices, including plug‐based or suture‐based techniques.

Surgical vascular access might prolong the procedure and is associated with major bleeding, blood transfusions, delayed mobilization and thus delayed discharge of the patients [3, 4, 5]. Vascular closure devices (VCD) can compensate for some of the disadvantages of surgical procedures but are technically complex and can also cause complications. In randomized trials of transfemoral aortic valve replacement (TAVR), the incidence of vascular complications is 6%–20% according to VARC‐3 standardized endpoint definitions [6, 7, 8, 9, 10, 11, 12, 13, 14, 15, 16], while a recent meta‐analysis reported major vascular complication to be as low as 3.4% for the femoral approach [17]. Today, most of these complications are due to failure of the arterial vessel closure technique, emphasizing the need for efficient and reliable VCDs [12].

The MANTA system, a plug‐based vascular closure device (PbVCD), is a collagen‐based tool specifically designed to close large‐bore arterial vascular access sites. The system has undergone a prospective multicenter evaluation to obtain CE certification and received pre‐market approval from the US Food and Drug Administration [18]. It enables percutaneous access side closure for TAVR, endovascular aneurysm repair (EVAR), and mechanical circulatory support (MCS) [19]. In particular for percutaneous microaxial flow pump (mAFP) supported CHIP procedures, operators are looking for effective and safe access site closure systems. Importantly, this setting might be different to the TAVR procedure since reversal of anticoagulation, for example, protamine, is typically not administered in patients who have just undergone coronary stent implantation.

There are limited data on the implementation of PbVCDs after the use of a mAFP in CHIP or cardiogenic shock. Therefore, we evaluated the safety and efficacy of the MANTA system in this patient cohort in a prospective multicenter registry.

## 2 Patients and Methods

### 2.1 Study Design and Setting

This prospective multicenter registry was conducted to evaluate the performance and safety of the PbVCD in CHIP or cardiogenic shock patients requiring mAFP. Due to the observational nature of this observation, no sample size analysis was performed, and the statistical analysis of the resulting data remained descriptive. A total of 73 patients were included during the enrollment phase of 12 months, starting in July 2018. The data was collected from four centers: University Hospital Aachen (UKA) (*n* = 30 patients), University Hospital Heidelberg (HD) (*n* = 5), Erasmus Medical Center (EMC) Rotterdam (*n* = 8), and the Heart Center Dresden (HCD) (*n* = 30).

Inclusion criteria were an indication for high‐risk coronary intervention or cardiogenic shock and selection of the transfemoral access route and an inner vessel diameter for access greater than 5 mm. Principal exclusion criteria were a vascular access site anatomy not suitable for percutaneous vascular closure, life expectancy of < 12 months because of noncardiac conditions, known aneurysm, necrosis or severe anomaly of the ascending aorta or aortic arch, mural thrombus in the left ventricle, pregnant or nursing patients

This clinical observation was conducted in accordance with the Declaration of Helsinki and in compliance with the study protocol. Local Ethic Committee (EC) approval was obtained. Following the closure procedure using PbVCD, the physician recorded treatment data and demographics and immediate adverse events as well as an assessment of the device performance. At patient discharge, in‐hospital and postoperative adverse events were re‐assessed and documented.

### 2.2 Patients

A screening log, documenting all mAFP patients, and the method of vessel closure used were maintained in each of the sites participating in the registry. Eligible for study inclusion were patients who were at least 18 years of age, nonpregnant and scheduled for treatment with the mAFP (ABIOMED IMPELLA 2.5 or CP) and an inner vessel diameter for access greater than 5 mm. All patients provided written informed consent before participating in the study.

### 2.3 Study Device and Procedures

In this study, we used the PbVCD Manta device from Teleflex. For this device, the 14 and 18 French system is indicated for femoral arterial closure after application of sheaths with a size from 12 to 25 French [20]. However, only the 14F Manta system was used in our patient cohort according to the instructions of use. Following the index procedure, baseline data, ultrasound and angiogram imaging data of the access site, procedural characteristics and outcomes were entered in an electronic database.

#### 2.3.1 Endpoints

The primary endpoint was the occurrence of vascular complications at the mAFP access site until discharge according to the Valve Academic Research Consortium‐3 (VARC‐3) definition [6]. Briefly, major vascular complications were defined as (1) arterial or venous injury (perforation, rupture, dissection, stenosis, ischemia, arterial, or venous thrombosis including pulmonary embolism, arteriovenous fistula, pseudoaneurysm, hematoma, retroperitoneal hematoma, infection) or compartment syndrome resulting in death, VARC type ≥ 2 bleeding, limb or visceral ischemia, or irreversible neurologic impairment. (2) Distal embolization (noncerebral) from a vascular source resulting in death, amputation, limb or visceral ischemia, or irreversible end‐organ damage. (3) Unplanned endovascular or surgical intervention resulting in death, VARC type ≥ 2 bleeding, limb or visceral ischemia, or irreversible neurologic impairment. (4) Closure device failure resulting in death, VARC type ≥ 2 bleeding, limb or visceral ischemia, or irreversible neurologic impairment.

Minor vascular complications were defined as (1) arterial or venous injury (perforation, rupture, dissection, stenosis, ischemia, arterial, or venous thrombosis including pulmonary embolism, arteriovenous fistula, pseudoaneurysm, hematoma, retroperitoneal hematoma, infection) or compartment syndrome *not* resulting in death, VARC type ≥ 2 bleeding, limb or visceral ischemia, or irreversible neurologic impairment. (2) Distal embolization (noncerebral) from a vascular source *not* resulting in death, amputation, limb or visceral ischemia, or irreversible end‐organ damage. (3) Unplanned endovascular or surgical intervention *not* resulting in death, VARC type ≥ 2 bleeding, limb or visceral ischemia, or irreversible neurologic impairment. (4) Closure device failure *not* resulting in death, VARC type ≥ 2 bleeding, limb or visceral ischemia, or irreversible neurologic impairment.

Secondary endpoints included the rate of procedure‐ or device ‐related bleedings, device‐ failures, and the time to hemostasis. Operators rated on the use of the MANTA system and the similarity compared to a SbVCD and ANGIOSEAL (Terumo Medical Corp.).

#### 2.3.2 Statistics

Categorical variables are expressed as numbers and percentage, continuous variables are expressed as the mean ± standard deviation. The statistical analysis was performed by VYSYO, using the software PASS 2024, V24.0.2, Released: February 28, 2024 (Central illustration 1).

**Central illustration 1:**
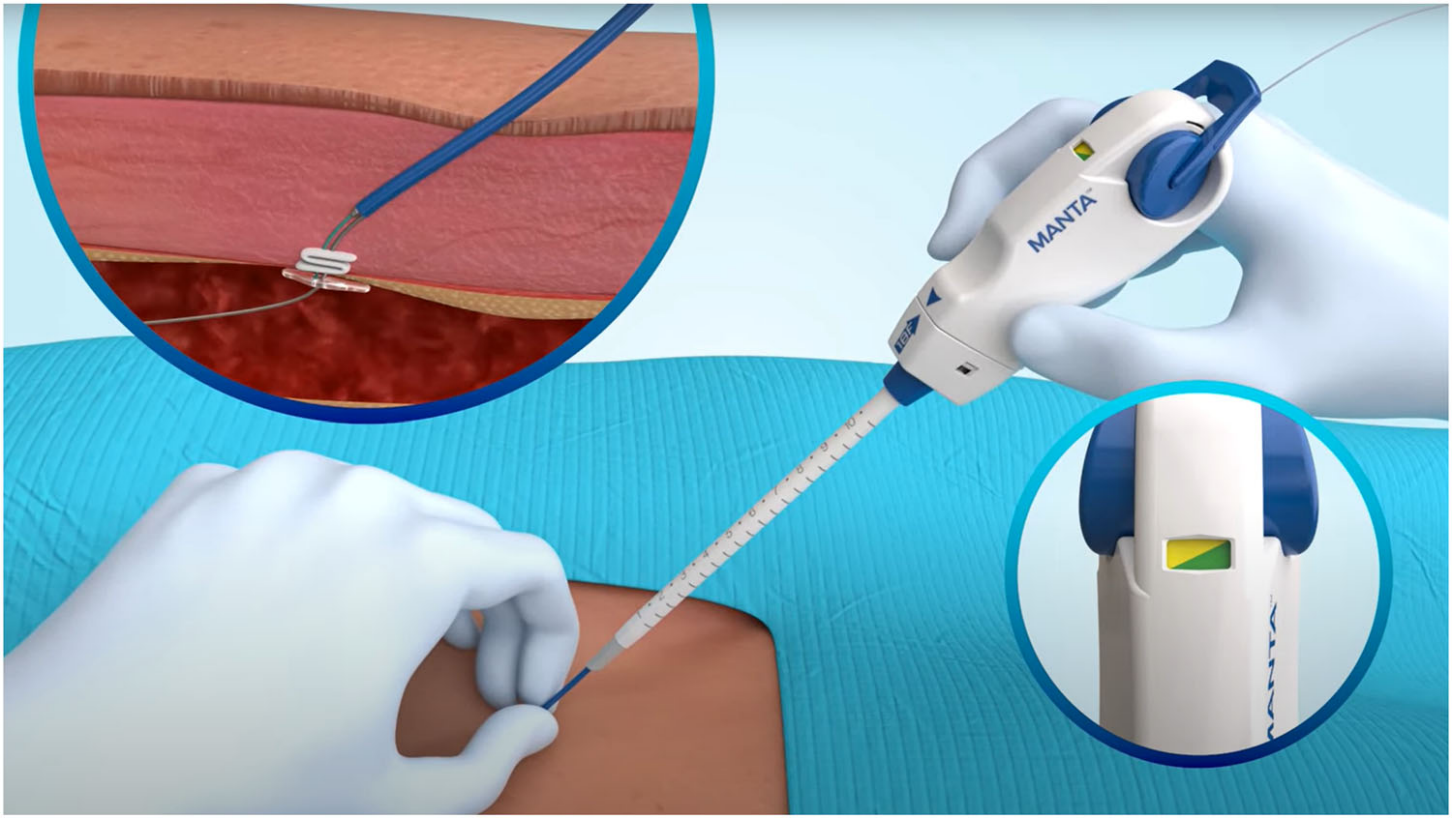
Closure of the vascular access usinga plug‐based system (Manta). [Color figure can be viewed at wileyonlinelibrary.com]

## 3 Results

### 3.1 Baseline and Procedural Characteristics

A total of 73 patients were included in this prospective registry. Baseline characteristics, procedural and access site data are summarized in Tables 1, 2, 3. The mean age was 78.7 ± 8.8years and 62 (85%) of the patients were male. Mean body mass index (BMI) was 26.6 ± 4.4 kg/m^2^ and mean left ventricular ejection fraction (LVEF) 41 ± 15%. Fifteen (20%) patients had peripheral artery disease. The indication for mAFP were high‐risk percutaneous coronary intervention (HRPCI) in 69 (94.5%) patients, acute myocardial infarction with cardiogenic chock (AMICS) in 2 (2.7%) patients, AMICS with HRPCI in 1 (1.4%) patient, and cardiogenic shock during a TAVR procedure (CS/TAVR) in 1 (1.4%) patient. Access was gained without imaging in only 7 (9.5%) patients while in the remaining ultrasound, angiography or a combination of both was used. Severe calcification of the access sit was evident in 7 (9.5) of patients.

**Table 1:** Patient characteristics.

Patient characteristics	*N* = 73
Age (years)	78.7 ± 8.8
Sex (male)	62 (85%)
Weight (kg)	78.9 ± 15.8
Body height (cm)	174 ± 9
Body mass index (kg/m^2^)	26.6 ± 4.4
Diabetes mellitus	26/73 (36.6%)
HbA1C	5.9 ± 0.8%
Smoker	33/73 (45.2%)
Hyperlipidemia	35/73 (47.9%)
Arterial hypertension	48/73 (65.8%)
Coronary artery disease	73/73 (100%)
Previous coronary intervention	42/73 (57.5%)
Previous artery bypass grafting	5/73 (6.8%)
Previous stroke	15/73 (20.5%)
Peripheral vascular disease	15 (20%)
Atrial fibrillation	25/73 (34.2%)
Renal disease (GFR < 60 mL/min)	35/73 (47.9%)
LVEF	41 ± 15%

*Note:* Numbers are mean ± standard deviation or numbers and frequencies.

**Table 2:** Procedure characteristics.

	*N*	%
Type of procedure		
HRPCI	69	94.5%
acuteMI&Shock, HRPCI	1	1.4%
acuteMI&Shock	2	2.7%
Shock/TAVR	1	1.4%
Total	73	100%

Abbreviations: acuteMI&Shock = acute myocardial infarction with cardiogenic shock; HRPCI = high risk PCI; Shock/TAVR = cardiogenic shock during TAVR procedure.

**Table 3:** Access site characteristics.

Access	*N*	%
Imaging of the access site before the procedure		
Angiogram and ultrasound	21	28.8%
Angiogram	65	87.8%
Ultrasound	23	31.1%
None	7	9.5%
Diameter of the common femoral artery (mm)	6.76 ± 0.49	
Level of calcification		
Mild	30	40.5%
Moderate	30	40.5%
Severe	7	9.5%
N/A	7	9.5%

### 3.2 Performance of the PbVCD

Table 4 summarizes the in‐hospital outcomes. Hemostasis was achieved without surgical intervention in all patients. Primary hemostasis without bleeding was achieved in 66 (90.4%) patients after the use of the PbVCD. Time to hemostasis was < 1 min for 55 (74.3%) of the patients and < 5 min for 64 (86.5%) of the patients. The maximum time to hemostasis was 20 min (in one patient).

**Table 4:** Outcome.

	*n*	%
Primary success^a^	71	97.3%
VARC‐3 access site complications		
None	66	90.4%
Minor	6	8.2%
Major	1	1.4%
Additional manual compression	16	21.6%
Time of manual compression (min)	7.6 ± 4.67 (minimum/maximum 1/15)	
Time to hemostasis (min)	2.41 ± 4.14 (minimum/maximum 1/20)	
Hemostasis within 1 min	55	74.3%
Hemostasis within 5 min	64	86.5%
Complications		
Hematoma^b^	7	9.5%
Delayed bleeding of access site^c^	3	5.4%
Access site infection	0	0%
Access site revision	0	0%
Retroperitoneal bleeding^d^	1	1.4%
Thromboembolic event	0	0%
Other device related events	0	0%
Fatal events during hospital stay	0	0%

a Two patients considered as nonprimary successful had immediate major oozing from the access site. One patient was very obese. Both patients were switched to manual compression.

b Hematoma (one patient with an additional retroperitoneal bleeding and Aneurysm spurium).

c Bleeding access site—(Oozing of blood: *n* = 1) (bleeding with compression needed *n* = 2).

d Retroperitoneal bleeding, self‐limiting, requiring blood cell transfusion *n* = 1.

The primary endpoint occurred in 7 (9.5%) patients. One patient experienced a major bleeding (VARC‐3 Type 2). This occurred immediately after device implantation with a self‐limiting retroperitoneal bleeding associated with a transfusion of two units of red‐packed blood cells. Minor bleedings (VARC‐3 Type 1) with hematoma formation occurred in six patients (8.2%) requiring manual compression with no further medical or interventional treatment. Three of these minor bleedings were delayed (*n*:3), occurring later than 1 h after primary hemostasis. No access site infection, surgical revision, thromboembolic event or limb ischemia or any other device‐related event was observed in the study group. (Table 4). The in‐hospital survival rate was 100%.

### 3.3 Operators Experience

Operators were already experienced with the application of the PbVCD. In 60 procedures (82.1%), the operators had already used the device more than 10 times. In five procedures (6.8%), the operator had used it between five and nine times and in nine procedures (12.3%), the operator had used it less than five times. No difference in AE rates was observed when considering the experience of the operators.

To assess operator satisfaction with handling, all operators were asked to rate the performance of the device during the procedure (Table 5). Operators rated the difficulty of using PbVCD in comparison to a SbVCD (Perclose ProGlide, Abbott Laboratories) as 2.4 ± 0.8 (maximum five) on a dimensionless scale from 1 to 10, ranging from *very easy* (1) to *very difficult* (10). The similarity of MANTA compared to ANGIOSEAL from Terumo was rated 2.6 ± 1.0 (maximum five) on a dimensionless scale from 1 (*similar*) to 10 (*different*).

**Table 5:** Device evaluation from the operator's perspective.

	Range: Very easy (1) to very difficult (10)
Difficulty of prelocate puncture	2.61 ± 1.14 (maximum: 7)
Difficulty of Manta sheath insertion	2.2 ± 0.82 (maximum: 4)
Difficulty of Manta device insertion	2.16 ± 0.84 (maximum: 4)
Difficulty of positioning and deployment of collagen seal	2.11 ± 0.8 (maximum: 4)
Difficulty to use of Manta compared to PerClose	2.41 ± 0.84 (maximum: 5)
	Range: Very similar (1) to very different (10)
Similarity of MANTA to ANGIOSEAL	2.62 ± 1.04 (maximum: 5)

## 4 Discussion

This multicenter, prospective, all‐comers registry of 73 patients receiving HRPCI with pMCS showed that the use of the MANTA System for vascular access site closure was safe and effective. Hemostasis was achieved in all patients with one (1.4%) major and six (8.4%) minor access site complications.

The potential benefit of mAFP support in HRPCI might be counterbalanced by bleeding and vascular complications and remains a concern for elective use in HR‐PCI, largely related to large‐bore vascular access. Such mAFP‐related vascular complications are linked to a significantly higher in‐hospital mortality, length of stay and cost limiting the use of prophylactic percutaneous circulatory support [21].

Our major access site complication rate of 1.4% is in line with the reported median rate of 2.6% (range 0%–8.3%) major vascular complications in the setting of mAFP‐supported HRPCI [22]. Moreover, rates of transfusion (in 1.4% of our cohort), major (1.4% in our cohort) and minor bleeding 8.4% in our cohort) are at the lower end of the reported events in other trials, for example, median rate of transfusion 5.4%, median rate of major bleeding 5.2% [22].

There is only limited data on the use of a specific VCD in the setting of mAFP‐supported HRPCI. A recent single‐center study assessed the PbVCD MANTA in 22 patients receiving mechanical circulatory support for HRPCI (68%) or cardiogenic shock (32%), with a majority receiving a mAFP. Technical success, defined as achievement of arteriotomy closure in absence of major bleeding or access site endovascular or surgical intervention, was reported in 96% of patients. Major access site bleeding occurred in 1 patient (4%) requiring endovascular balloon tamponade [23].

More evidence has been developed in the setting of large bore access in the context of transcatheter aortic valve implantation (TAVI). It's noteworthy to state that this population differs from the setting of HRPCI or cardiogenic shock [24] in many factors, in particular with regard to reversal of anticoagulation, for example, administration of protamine at the end of a TAVI procedure. Comparing the PbVCD MANTA with SbVCD Perclose Proglide after TAVI in an observational study, Dumpies et al. [25] reported significantly less access site‐related complications in 578 patients using PbVCD compared to SbVCD (10.7% vs. 16.60%, *p* = 0.048). Moriyama et al. [26] reported less vascular complications (14% with PbVCD vs. 21% with SbVCD, *p* = 0.21) and bleeding complications (18% with PbVCD vs. 33% with SbVCD, *p* = 0.01) in 111 propensity score‐matched patients. In the randomized CHOICE‐CLOSURE study [27], access site related major and minor vascular complications occurred more frequently after PbVCD (19.4%) compared to SbVCD (12.0%; RR: 1.61; 95% CI: 1.07–2.44, *p* = 0.029), mainly driven by pseudoaneurysm and local hematoma. Importantly, more than 98% of patients received half‐ or full‐dose protamine. In a recently published meta‐analysis [20] and randomized clinical trials [28, 29], the PbVCD and SbVCD (including the devices ProGlide, MANTA, and Prostar XL) had no significant difference in access site‐related vascular complications.

A pilot RCT comparing the MANTA VCD versus ProGlide [29] showed similar complication rates and revealed that suture‐based closure required more often additional closure devices, whereas MANTA numerically needed more covered stents and surgical bailouts.

It can be assumed that the patient's constitution and vascular characteristics influence the complication rate more than the choice of closure device. Pronounced vascular calcification and BMI have already been identified as risk factors for complications at the access site [30, 31]. Similarly, the ratio of Sheath/CFA ≥ 0.9 (diameter of the Sheath to the femoral artery) was recognized as a significant complication parameter for bleeding at the vascular access site [32]. It is also striking that there is a huge variety in the different studies regarding the rate of vascular complications, so that inter‐study comparisons seem to be difficult.

To better compare different VCD, controlled randomized studies are required to study the true impact on vascular and bleeding events as well as to identify patient and/or vessel characteristics that predict the success of closure of each device technology.

The technical similarity of the MANTA system to the ANGIOSEAL System, which is widely used in smaller punctures after cardiac catheterization via the femoral artery, explains the assessment of easy handling. The users in our study confirmed efficient and safe use, regardless of their level of experience with the PbVCD.

## 5 Conclusion

In this study the PbVCD was found to be a safe, efficient and easy‐to‐use vascular closure system for large‐bore access. Hemostasis was achieved in all patients and complication rates were comparable to the rates reported in current literature.

## Ethics Statement

This study was approved by the Medical Ethics Committee of RWTH Aachen University, reference number EK 170/18.

## Conflicts of Interest

N.M. reports personal fees from Edwards Lifesciences, Medtronic, Biotronik, Novartis, Sanofi Genzyme, AstraZeneca, Pfizer, Bayer, Abbott, Abiomed, B. Braun, and Boston Scientific, outside the submitted work.

## Data Availability

The data that support the findings of this study are available on request from the corresponding author. The data are not publicly available due to privacy or ethical restrictions.
